# Surfing the vegetal pole in a small population: extracellular vertical transmission of an 'intracellular' deep-sea clam symbiont

**DOI:** 10.1098/rsos.160130

**Published:** 2016-05-18

**Authors:** Tetsuro Ikuta, Kanae Igawa, Akihiro Tame, Tsuneyoshi Kuroiwa, Haruko Kuroiwa, Yui Aoki, Yoshihiro Takaki, Yukiko Nagai, Genki Ozawa, Masahiro Yamamoto, Ryusaku Deguchi, Katsunori Fujikura, Tadashi Maruyama, Takao Yoshida

**Affiliations:** 1 Department of Marine Biodiversity Research, Japan Agency for Marine-Earth Science and Technology (JAMSTEC), 2-15 Natsushima, Yokosuka, Kanagawa 237-0061, Japan; 2 Department of Subsurface Geobiological Analysis and Research, Japan Agency for Marine-Earth Science and Technology (JAMSTEC), 2-15 Natsushima, Yokosuka, Kanagawa 237-0061, Japan; 3 Research and Development Center for Marine Biosciences, Japan Agency for Marine-Earth Science and Technology (JAMSTEC), 2-15 Natsushima, Yokosuka, Kanagawa 237-0061, Japan; 4 Graduate School of Marine Science and Technology, Tokyo University of Marine Science and Technology, 4-5-7 Konan, Minato-ku, Tokyo 108-8477, Japan; 5 Marine Works Japan, Ltd, 3-54-1 Oppamahigashi, Yokosuka, Kanagawa 237-0063, Japan; 6 Department of Chemical and Biological Sciences, Japan Women's University, 2-8-1 Mejirodai, Bunkyo-ku, Tokyo 112-8681, Japan; 7 Core Research for Evolutional Science and Technology (CREST), Japan Science and Technology Agency, Gobancho, Chiyoda-ku, Tokyo 102-0076, Japan; 8 Department of Marine Biosciences, School of Marine Biosciences, Kitasato University, 1-15-1 Kitasato, Minami-ku, Sagamihara, Kanagawa 252-0373, Japan; 9 Department of Biology, Miyagi University of Education, Aoba-ku, Sendai, Miyagi 980-0845, Japan

**Keywords:** endosymbiosis, deep-sea clam, vertical transmission, population size

## Abstract

Symbiont transmission is a key event for understanding the processes underlying symbiotic associations and their evolution. However, our understanding of the mechanisms of symbiont transmission remains still fragmentary. The deep-sea clam *Calyptogena okutanii* harbours obligate sulfur-oxidizing intracellular symbiotic bacteria in the gill epithelial cells. In this study, we determined the localization of their symbiont associating with the spawned eggs, and the population size of the symbiont transmitted via the eggs. We show that the symbionts are located on the outer surface of the egg plasma membrane at the vegetal pole, and that each egg carries approximately 400 symbiont cells, each of which contains close to 10 genomic copies. The very small population size of the symbiont transmitted via the eggs might narrow the bottleneck and increase genetic drift, while polyploidy and its transient extracellular lifestyle might slow the rate of genome reduction. Additionally, the extracellular localization of the symbiont on the egg surface may increase the chance of symbiont exchange. This new type of extracellular transovarial transmission provides insights into complex interactions between the host and symbiont, development of both host and symbiont, as well as the population dynamics underlying genetic drift and genome evolution in microorganisms.

## 1. Introduction

Across animals and plants, symbiotic associations with beneficial microbes confer adaptive fitness on the host. Maintenance of symbiotic associations through evolutionary time relies on the stable transmission of the symbionts from generation to generation. Thus, symbiont transmission is a key event for understanding the processes underlying symbiotic associations and their evolution. Symbiotic microbes can be transmitted horizontally (between contemporary hosts or through reinfection by symbionts as free-living forms), vertically (directly from parent to offspring, often via gametes), or through a mixed mode (a combination of the two transmission mechanisms) [1]. Despite their biological significance, mechanisms of symbiont transmission are not well understood.

In chemosynthetic ecosystems, a wide variety of animals have acquired the ability to live on inorganic carbon sources by establishing symbioses with chemoautotrophic bacteria. Deep-sea vesicomyid clams, including the genus *Calyptogena*, are endemic and dominant members in deep-sea chemosynthesis-based communities [2]. They harbour sulfur-oxidizing intracellular symbiotic bacteria, belonging to Gram-negative gamma-proteobacteria, in their gill epithelial cells [3]. Because their digestive tracts are not functional, the clams depend on their symbionts for nutrition [4]. In such obligate host–symbiont associations, vertical transmission is a secure process for successful symbiont inheritance. Across various taxa, routes of vertical transmission differ among symbiotic systems. Intracellular obligate symbionts are generally unable to survive outside the host cells and are transmitted transovarially, whereas many extracellular symbionts are transmitted by post-hatch mechanisms that require the symbionts to survive outside the hosts for a part of their life cycle [5,6]. It has been proposed that the symbionts of vesicomyid clams are vertically transmitted via eggs [7–9], with a small transmission symbiont population size, which is an important driving force for reductive genome evolution based on the genetic drift [3]. Additionally, the occasional horizontal acquisition of the symbiont has been suggested to occur [10]. However, the question of how the symbiont associates with the spawned eggs has remained unanswered, and the population size of the symbionts transmitted via eggs has not been determined.

To address these issues, we have established a method for artificial on-board induction of spawning of the deep-sea clam, *Calyptogena okutanii*, and investigated the localization of the symbiont associating with spawned eggs as well as ovarian oocytes, cell number of the symbiont carried by a single egg, and genomic copy number in a single symbiont cell. Our results show a unique localization of the symbiont on the outer surface of the egg at the vegetal pole, and reveal the population size of the symbiont transmitted via eggs. Based on the findings, we discuss the transmission mode of the vesicomyid clam symbiont, its correlation with host development, and the evolution of the symbiont genome.

## 2. Material and methods

### 2.1. Animal sampling

All *C. okutanii* were collected using the ROV *Hyper-Dolphin*, operated by the R/V *Natsushima* of the Japan Agency of Marine-Earth Science and Technology. Sampling sites were Off Hatsushima Island seep sites in Sagami Bay at a depth of 856 m (35°00.954′ N, 139°13.337′ E, Dive#1293) during cruise NT11-09 (15–26 June 2011); 857 m (35°00.965′ N, 139°13.324′ E, Dive#1508) during cruise NT13-07 (2–10 April 2013); 857 m (35°00.948′ N, 139°13.310′ E, Dive#1641), 949 m (35°00.924′ N, 139°13.426′ E, Dive#1643) and 860 m (35°00.966′ N, 139°13.329′ E, Dive#1644) during cruise NT14-05 (2–8 April 2014) and the Iheya North hydrothermal vent field in the mid-Okinawa Trough at a depth of 1055 m (27°47.403′ N, 126°54.020′ E, Dive#1769 and #1773) during cruise NT15-02 (11–27 January 2015). The collected clams were either kept in aquarium tanks at 4°C for spawning induction as described below or immediately dissected on-board. The gill, gonad and foot were cut out using a disposable scalpel and frozen immediately in liquid nitrogen or fixed as follows. For *in situ* hybridization (ISH) analysis and haematoxylin–eosin (HE) staining, the gonads (approx. 45 × 25 × 10 mm) were cut into small pieces and fixed in 4% paraformaldehyde in 1× phosphate-buffered saline (PBS) for 16 h at 4°C, followed by stepwise dehydration in an ethanol series. For transmission electron microscope (TEM) observation, the gills and gonads were cut into small pieces and fixed in 2.5% glutaraldehyde in seawater filtered with a 0.2 µm filter unit (Nalgene, Rochester, NY, USA; filtered seawater, FSW) at 4°C. The species of clam was identified by the multiplex-PCR identification method described previously [11] or by sequencing of the mitochondrial cytochrome oxidase subunit I gene using DNA extracted from the foot with DNeasy Blood and Tissue Kit (Qiagen, Hilden, Germany) as a template.

### 2.2. Spawning induction and egg sampling

To induce spawning, the clams kept in aquarium tanks were injected on-board in the foot with 0.2–0.4 ml of 50–100 µM 5-hydroxytryptamine (5-HT) (Nakarai, Kyoto, Japan). Immediately after injection, each clam was placed individually in a plastic container, holding about 2 l of seawater at 4°C (electronic supplementary material, figure S1*a*) and observed for spawning (electronic supplementary material, figure S1*b*). All spawning experiments lasted several hours, after which eggs were collected and washed more than five times with FSW at 4°C. For quantitative polymerase chain reaction (qPCR) analysis, a 1 µl volume of an egg and FSW was transferred into a 0.2 ml sample tube and frozen immediately in liquid nitrogen. For whole-mount *in situ* hybridization (WISH), eggs were fixed with 4% paraformaldehyde in 0.1 M MOPS (pH 7.5) and 0.5 M NaCl for 16 h at 4°C. After equilibration with PBS containing 0.1% Tween 20 (PBST), the eggs were treated with 2 µg ml^−1^ proteinase K (Takara, Shiga, Japan) in PBST for 30 min at 37°C. They were refixed with 4% paraformaldehyde in PBST at room temperature for 1 h, and washed with PBST. Because eggs burst in a solution containing alcohol, they were stored in a solution containing 50% formamide (FA), 4 × 0.6 M NaCl and 60 mM sodium citrate, 50% dextran sulfate sodium, 0.1 mg ml^−1^ torula yeast RNA (Sigma-Aldrich, St Louis, MO, USA) and 0.1% sodium dodecyl sulfate (SDS) at 4°C. For TEM observations, eggs were fixed in 2.5% glutaraldehyde in FSW at 4°C. For DAPI staining, eggs were fixed in 1% glutaraldehyde in FSW for 16 h at 4°C, and stored in FSW at 4°C. After spawning experiments, the 5-HT-injected clams were dissected for sexual identification, easily recognized by morphological observation of gonads, and the foot was frozen immediately in liquid nitrogen for species identification as described above.

### 2.3. Whole-mount *in situ* hybridization

The stored eggs were soaked into hybridization buffer (20% FA, 0.9 M NaCl, 20 mM Tris–HCl pH 7.5, 5 mM EDTA, 0.01% SDS) twice for 10 min and hybridized in hybridization buffer containing 0.5 µM of probe at 46°C overnight. Probe sequence for WISH was the same as Cok 16S_1 (5′-AGCTTCGCCACTAAAGGGTACCCCC-3′), which was designed to be specific to *16S rRNA* gene of the *C. okutanii* symbiont [12], and its 5′ end was labelled with digoxigenin (DIG). For the negative control, the ‘No-bind probe’ (5′-CCTAGTGACGCCGTCGAC-3′) [13] labelled with DIG was used. After hybridization, excess probe was washed twice in washing solution containing 0.215 M NaCl, 20 mM Tris–HCl pH 7.5, 5 mM EDTA and 0.01% SDS for 30 min at 48°C. After washing in PBST twice for 15 min, the eggs were incubated in 0.5% blocking reagent (Roche, Basel, Switzerland) in PBST for 30 min and then incubated overnight in a 1/2000 volume of anti-DIG-AP (Roche) in PBST containing 0.5% blocking reagent at 4°C. The specimens were washed four times in PBST for 20 min and twice in buffer containing 0.1 mM Tris pH 9.5, 0.05 mM MgCl_2_, 0.1 mM NaCl, 0.1% Tween 20 (TMNT), then incubated in 1× NBT/BCIP solution (Roche) including 2 mM levamisole in TMNT. After the staining procedure was complete, the eggs were washed twice in PBST. Because all eggs floated with animal pole up in PBST, eggs were embedded in 1% agarose gel (low gelling temperature, Sigma-Aldrich) in PBS to observe the signals at the vegetal pole with a stereomicroscope. For DAPI staining of WISH samples, the eggs were soaked in Vectashield with DAPI (Vector Laboratories, Burlingame, CA, USA). Images were captured using an Olympus SZX16 stereomicroscope or an Olympus IX73 microscope equipped with an Olympus DP73 camera.

### 2.4. *In situ* hybridization and haematoxylin–eosin staining

The gonads were embedded in paraffin and sliced with a microtome into 8 µm-thick serial sections measuring about 6 × 4 mm in area size. More than 30 serial sections of the ovary were prepared for one adult host individual. The sections were de-waxed by three successive washes in xylene for 10 min each followed by absolute ethanol for 5 min each. Rehydration was accomplished by stepwise transfer into 70%, 50%, and 30% ethanol and followed by transfer into PBS for 10 min twice. Using these sections, HE (Muto Pure Chemicals, Tokyo, Japan) staining was performed according to the manufacturer's instructions. For ISH, the sections were treated with 1 µg ml^−1^ proteinase K (Takara) in PBS for 10 min at 37°C, refixed with 4% paraformaldehyde in PBS at room temperature for 20 min and washed with PBST. Then, the sections were hybridized in hybridization buffer containing 0.5 µM Cok 16S_1 probe labelled with DIG for 2 h at 46°C. For negative control, ‘No-bind probe’ labelled with DIG was used. After hybridization, excess probe was washed twice in washing solution (see above) at 48°C. After washing in PBST twice for 20 min, the sections were incubated in 0.5% blocking reagent (Roche) in PBST for 30 min and then incubated overnight in a 1/2000 volume of anti-DIG-AP (Roche) in PBST containing 0.5% blocking reagent at 4°C. The sections were washed three times in PBST for 20 min and once in TMNT buffer, and then incubated in 0.1 × NBT/BCIP solution (Roche) including 2 mM levamisole in TMNT. After the staining procedure was complete, the sections were washed twice in PBS and mounted in Vectashield with DAPI (Vector Laboratories). Images were captured using an Olympus IX73 microscope equipped with an Olympus DP73 camera.

### 2.5. Polymerase chain reaction for detecting the symbiont in the gonads

Total DNA was extracted from the ovaries of three female host individuals and the testes of three male host individuals using a DNeasy Blood and Tissue Kit (Qiagen). The PCR primers used in this study were designed to amplify a 1508-bp sequence in the *16S rRNA* gene of *C. okutanii* symbiont (accession no., NC_009465) (27F, 5′-AGAGTTTGATCCTGGCTCAG-3′ and 1492R, 5′-GGTTACCTTGTTACGACTT-3′) [14]. For PCR amplification, *LA Taq* DNA polymerase (Takara) was used with an initial denaturation phase of 96°C for 2 min, followed by 30 cycles of 96°C for 20 s, 56°C for 10 s and 72°C for 1 min 30 s, using 20 ng of total DNA extracted from the gonads of each individual as the template in a reaction volume of 25 µl. The sequences of PCR products from the ovary DNA were confirmed by DNA Sanger sequencing and matched 100% to the *C. okutanii 16S rRNA* gene sequence.

### 2.6. Transmission electron microscope observation

For egg sections, fixed eggs were washed with filtered (pore size = 0.2 µm) artificial seawater (Rohto Marine, Iwaki, Tokyo, Japan) (FASW) and post-fixed with 2.0% osmium tetroxide dissolved in FASW for 2 h at 4°C. After washing with 8.0% sucrose aqueous solution, the conductive staining was performed by incubating 0.5% thiocarbohydrazide (Thermo Fisher Scientific, Waltham, MA, USA) aqueous solution for 30 min and 1.0% osmium tetraoxide aqueous solution for 1 h at 4°C. The samples were washed, dehydrated in a graded series (30, 50, 70, 80, 90 and 100%) of *N*,*N*-dimethylformamide, cleared in *n*-butyl-glycidyl-ether (Nisshin EM, Tokyo, Japan) and embedded in Quetol 651 (Nisshin EM). Ultra-thin sections (60 nm thickness) were cut with a diamond knife on an Ultracut S ultra-microtome (Leica Microsystems, Wetzlar, Germany), stained with 2.0% uranyl acetate solution and 2.0% lead citrate solution, and observed using a Tecnai G^2^ 20 electron microscope (FEI, Hillsboro, OR, USA) operated at 120 kV. Thin sections of the ovary and gill were prepared as described above with modification in the dehydration process by using a graded series (30, 50, 70, 80, 90 and 100%) of ethanol.

### 2.7. Quantitative polymerase chain reaction

The qPCR primers used in this study were designed to amplify a 98-bp fragment of *dnaA* of the *C. okutanii* symbiont, which is a single-copy gene (accession no., NC_009465) (dnaA-qF, 5′-AATGGCAATGTTTATTTGCTATGAAC-3′; dnaA-qR, 5′-TAGCGTGTAATACAGTGGAATGATCTC-3′), and a normal minor groove binding (MGB) TaqMan probe (5′-TTGGTAAACACTTCGGAAAT-3′) was designed to hybridize to this qPCR product. The sequence specificity of the primers and probe was confirmed by comparison with all available sequences on the GenBank database via BLASTN search (http://blast.ncbi.nlm.nih.gov/Blast.cgi), and we confirmed that no other sequences were detectable using our set of qPCR primers and probes in the database including the genome sequence of the *C. okutanii* symbiont. The qPCR primers and probe were obtained from Applied Biosystems (Thermo Fisher Scientific). Triplicates of a 10-fold dilution series (from 1 × 10^–2^ to 1 × 10^–6^ molecules) of the PCR fragment encompassing the region targeted by each qPCR primer set was used as a template to produce a calibration curve for absolute quantification. The PCR primers used for template preparation to produce calibration curves were as follows: StdF, 5′-GGGAGCACTTCTTAAACTCAAAGC-3′; StdR, 5′-CATGTGCAAGGATAGGTAATGTTTG-3′.

The qPCR analysis was performed on an Applied Biosystems 7300 Real-Time PCR System (Thermo Fisher Scientific) in 25 µl reaction mixtures containing 12.5 µl of Ampdirect Plus (Shimazu), 0.2 µM concentrations of each primer, 0.2 µM TaqMan probe, 1× Rox Reference Dye (Thermo Fisher Scientific), 0.625 units of BIOTAQ™ HS DNA Polymerase (Shimazu) and 1 µl volume of an egg with FSW. The reaction consisted of 2 min at 50°C, 10 min at 95°C and 40 cycles of 95°C for 15 s and 60°C for 1 min. No-template controls (NTC) with 1 µl of FSW, in which eggs were kept until harvesting, were run under the same conditions, and produced no threshold cycle value (Cq). To address the technical repeatability and reproducibility, we quantified *dnaA* in six respective eggs from five different host individuals. The sequences of amplicons were confirmed by DNA Sanger sequencing and matched 100% to the *C. okutanii dnaA*. In BLASTN searches with the amplicon sequences, the sequence matched no other sequence except for that of *dnaA* of *C. okutanii*. Quantification analysis was performed by using SDS, v. 1.3.1 software (Thermo Fisher Scientific). The values acquired with the calibration curves were computed reflecting PCR amplification efficiency (efficiency = 95.55%, slope = −3.433, *y*-intercept = 40.67, *R*^2^ = 1.000; electronic supplementary material, table S1). Statistical analysis was performed using Microsoft Office Excel for Mac 2011 (Microsoft, Redmond, WA, USA). All qPCR analyses followed the MIQE guidelines [15].

### 2.8. Cell number counting and video-intensified microscope photon-counting system analysis

Eggs were fixed in 1% glutaraldehyde in FSW for 16 h at 4°C, and stored in FSW at 4°C. Fixed eggs were stained with DAPI on a cover glass in 6 µl of 1 µg ml^−1^ of DAPI (Roche) and 1% glutaraldehyde in buffer containing 20 mM Tris–HCl pH 7.5, 0.5 mM EDTA, 1.2 mM spermidine, 7 mM 2-mercaptoethanol and 0.4 mM phenylmethylsulfonyl fluoride (TAN buffer) [16], and another cover glass was pressed down gently on the egg. While the peripheral structure of the egg was partially broken due to the pressure, counts were still possible. Observation by mounting the egg between two cover glasses effectively reduced the influence of the autofluorescence. To count the number of the symbiont cells, images were captured using a Nikon A1RMP confocal scanning system with some *z*-axis scanning as necessary, and the spots of the DAPI signal were counted manually using NIS-Elements software (Nikon, Tokyo, Japan). Number of symbiont cells was counted on three eggs from each of five host individuals (electronic supplementary material, table S1). For analysis of DNA content by fluorimetry, fluorescent intensities from 46 individual symbiont cells were quantified using a video-intensified microscope photon-counting system (VIMPCS; Hamamatsu Photonics, Shizuoka, Japan) connected to an Olympus BX51 microscope (Olympus, Tokyo, Japan) as described previously [17,18]. Genomic copy number was estimated based on the calculated DNA content and the genome size of the symbiont (1.02 Mbp [3]). For standardization of the fluorescence intensity of specimens, *Escherichia coli* (temperature-sensitive *dnaC* mutant strain defective in the initiation of chromosome replication: ME7916 derived from PC2 [19]) was grown in LB including 50 µg ml^−1^ thymine for 16 h at 30°C and 2 h at 42°C, then collected and fixed with 1% glutaraldehyde in TAN buffer (electronic supplementary material, table S1).

## 3. Results

### 3.1. Unique localization of the symbiont associating with *Calyptogena* eggs

In a previous study, spawning of the *Calyptogena* clam was induced *in situ* by artificially increasing the ambient water temperature [20]. Since this method requires specialized instruments and considerable effort to collect the eggs, we sought to develop a method to induce spawning on-board, and found that injection of 5-HT into the foot of the clam was very simple and effective for spawning (electronic supplementary material, figure S1*a*,*b*). The on-board spawned unfertilized eggs were 210–250 µm in diameter (electronic supplementary material, figure S1*c*) and buoyant in seawater.

**Figure 1.**
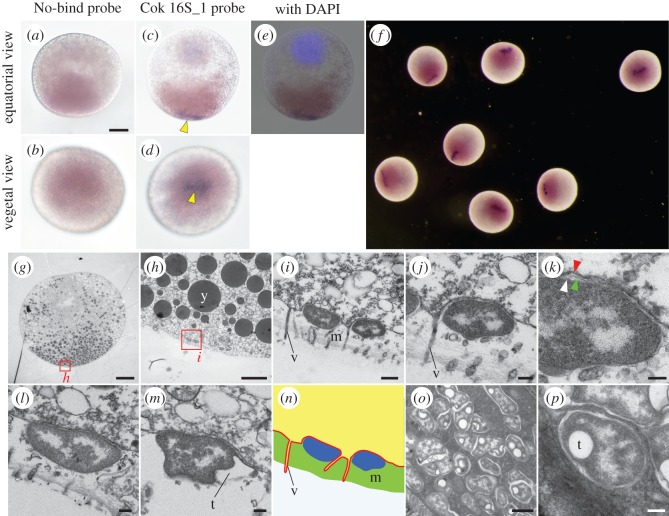
Localization of the *C. okutanii* symbiont associating with spawned eggs. (*a–f*) WISH on spawned eggs, using No-bind probe (*a*,*b*) and Cok 16S_1 probe (*c–f*), with an image of egg nucleus stained with DAPI merged (*e*). The signals for Cok 16S_1 probe (indicated with yellow arrowheads in *c* and *d*) are located opposite from the localized egg nucleus, where yolk pigmentation is observed, i.e., the vegetal pole. (*f*) Sibling eggs showing the symbiont signals at the vegetal pole. (*g–m*) TEM images of the symbiotic bacteria of *C. okutanii* on eggs. Animal side is up. (*g–k*) High magnification images from TEM demonstrating that the symbionts are attached extracellularly to the egg plasma membrane. Red rectangles in (*g*) and (*h*) correspond to the locations of the following magnified images. White, green and red arrowheads in (*k*) indicate bacterial inner membrane, bacterial outer membrane and host cell membrane, respectively. (*l*,*m*) TEM images of other symbiont cells on other eggs. (*n*) A schematic drawing showing localization of the symbiont on the egg surface. Red line indicates egg cell membrane. Yellow, dark blue and green areas indicate egg cytoplasm, symbiont and vitelline membrane, respectively. (*o*,*p*) TEM images of the symbionts in the gill. m, vitelline membrane; t, electron transparent vesicle; y, yolk granule; v, microvilli. Scale bars in *a* (applicable to *a–e*) and (*g*) 50 µm; (*h*) 5 µm; (*i*) 500 nm; (*j*, *l*, *m*) and (*p*) 200 nm; (*k*) 100 nm; (*o*) 1 µm.

To investigate the localization of the symbiont in the spawned eggs, we conducted WISH analysis using a specific probe designed for the symbiont of *C. okutanii* [12]. To avoid the strong autofluorescence of the yolk, and to obtain a complete view of the egg, we employed a probe labelled with DIG, and the conventional NBT/BCIP chromogenic staining. The signal for the symbiont was detected on the surface of the egg in an approximately oval-shaped cluster, measuring approximately 100 µm × 20–50 µm, whereas no signal was detected when using the negative control probe (figure 1*a–f*). The WISH signal was detected opposite the observed egg nucleus, on the surface where yolk pigmentation was observed, i.e. the vegetal pole (figure 1*c–f*).

In transmission electron micrographs, one end of the egg had numerous electron-lucent lipid droplets and the other end had crowded electron-dense yolk granules (figure 1*g*). The germinal vesicle was not observed, indicating the egg was mature (figure 1*g*). The egg was enclosed by a non-cellular vitelline membrane, and microvilli were observed on the plasma surface of the egg, as observed in other molluscs [21] (figure 1*h–j*). Prokaryotic cells with a typical Gram-negative cell envelope were found on the outer surface of the egg plasma membrane at the area where numerous yolk granules were observed (figure 1*g–m*). This localization was consistent with the results of WISH (figure 1*c–f*). Cell shape was mainly oval, but sometimes irregular, and average cell size was 1.4 (s.d. = 0.4; *n* = 50) × 0.7 (s.d. = 0.2; *n* = 50) μm (electronic supplementary material, table S1). Electron transparent vesicles, which might be sites of elemental sulfur storage [22,23], were often observed in the symbiont cells located in the gill (figure 1*o*,*p*), whereas they were rarely found in those on the egg (figure 1*i–m*). Despite extensive observation, we did not find bacterial cells inside the egg. Thus, *C. okutanii* bacterial symbionts were only found on the outer surface of the plasma membrane at the vegetal pole of the spawned eggs.

**Figure 2.**
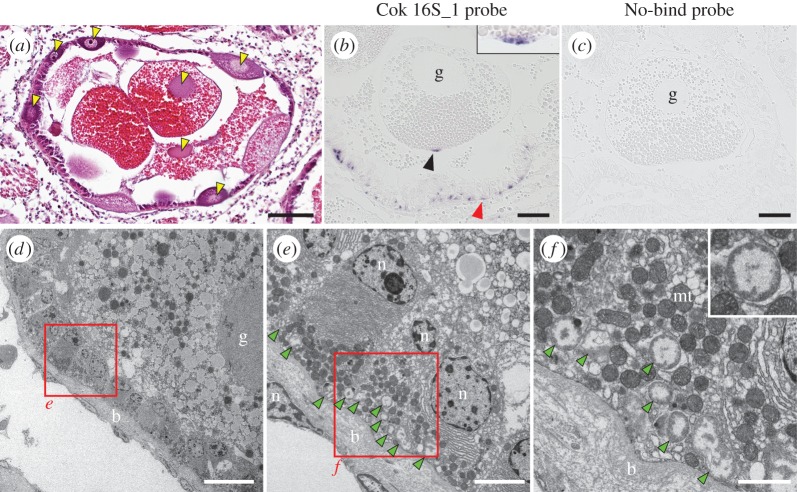
Localization of the *C. okutanii* symbiont in the ovary. (*a*) Image of haematoxylin–eosin (HE) staining showing typical structure of the ovarian acinus, which is composed of epithelial acinal wall cells and the oocytes in various maturation stages. The oocytes are recognized by huge, conspicuous nuclei, germinal vesicles (yellow arrowheads). (*b* and *c*) Localization of the symbiont in the ovary analysed by ISH using Cok 16S_1 probe (*b*) and No-bind probe (*c*). As in the spawned eggs, signals are seen at the vegetal pole (black arrowhead in *b*). Signals are also observed in the basal side of acinal wall cells (red arrowhead in *b*). Inset in (*b*) shows higher magnification of the vegetal pole. g, germinal vesicle. (*d–f*) A series of TEM images of the ovary using increasing levels of magnification. Red rectangles in (*d*) and (*e*) correspond to the locations of the following magnified images. Green arrowheads in (*e*) and (*f*) indicate the symbionts. The symbionts enclosed by a vacuolar membrane are seen in the basal side of the acinal wall cells along with an abundance of mitochondria. Inset in (*f*) shows higher magnification of a symbiont cell. Electron transparent vesicles are not observed in the symbiont cells. b, basement membrane; g, germinal vesicle; n, nucleus; mt, mitochondria. Scale bars in (*a*) 100 µm; (*b*,*c*) 50 µm; (*d*) 20 µm; (*e*) 5 µm; (*f*) 2 µm.

It is possible that WISH signals were not detected in the egg cytoplasm due to reduced reagent penetration into the egg. It is also possible that symbiotic bacteria were present on the surface of the eggs due to contamination, given that the eggs were collected from seawater in which the host was cultured. Therefore, we investigated localization of the symbiont in the *C. okutanii* gonads, by section ISH analysis using the same probe as in WISH. *C. okutanii* is a gonochoristic species, having a male or female gonad in separate adult individuals, and no evidence for hermaphroditism was found. In the male testis, the symbiont was not detected (electronic supplementary material, figure S2). The female ovary consisted of a cluster of acini, which was composed of epithelial acinal wall cells, also called follicle cells, and oocytes in various maturation stages (figure 2*a*). Oocytes were recognized by huge, conspicuous nuclei, the germinal vesicles (figure 2*a–c*). In the ovary, ISH signals were detected in a small part of the periphery of the oocyte, opposite the germinal vesicles (figure 2*b*). Despite extensive observation of more than 30 serial sections of the ovary, which included several hundred oocytes per section and covered a whole oocyte in terms of thickness, we did not detect signals inside the oocyte. Symbionts were also detected in the basal side of the acinal wall cells in both ISH and TEM observations (figure 2*b*,*d–f*). Electron transparent vesicles were not observed in the symbiont cell in the acinal wall cells (figure 2*f*). Thus, we conclude that the endosymbiotic bacteria of *C. okutanii* are definitely associated with the host's egg and are inherited transovarially, but they are attached extracellularly on the outer surface of the egg plasma membrane, exclusively at the vegetal pole, forming an oval-shaped cluster. At this moment, however, we cannot completely exclude the possibility that the symbionts also occur in the egg cytoplasm.

### 3.2. Population size and genomic copy number of the *Calyptogena okutanii* symbiont transmitted via eggs

**Figure 3.**
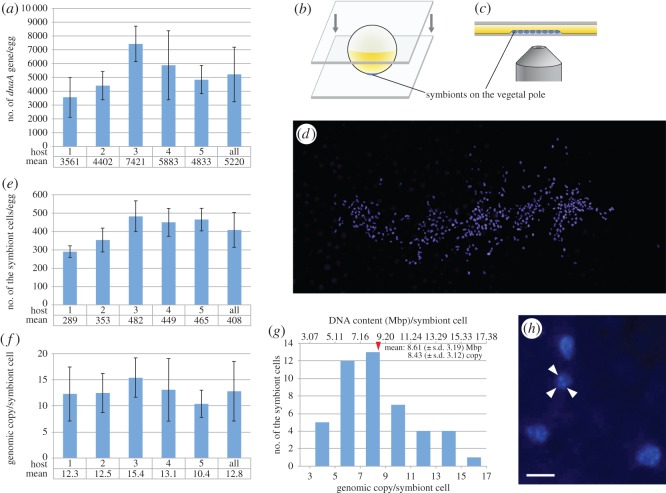
Population size and genomic copy number of the *C. okutanii* symbiont transmitted via eggs. (*a*) Means of the amount of symbiont genomic DNA per egg from five different host individuals analysed by qPCR. Quantity of *dnaA* gene was measured in six eggs from each host individual. The rightmost bar is the mean of the total (30 eggs) from five hosts. Error bar, standard deviation. (*b–d*) Preparation of samples for counting of the symbiont cell numbers and for VIMPCS analysis. Eggs were stained with DAPI between two pieces of cover glass. The original cluster of the symbiont located in an oval shape on the egg was preserved between the plasma membrane of the egg and the cover glass, and could be observed from either side of the two cover glasses (*c*,*d*). (*e*) Mean number of the symbiont cells stained with DAPI per egg from five hosts. Number of symbiont cells was counted on three eggs from each host individual. The rightmost bar is the mean number of the total (15 eggs) from five host individuals. Error bar, standard deviation. (*f*) Mean genomic copy numbers per symbiont cell calculated from *a* and *e*. The rightmost bar is the overall mean value. Error bars, standard deviation. (*g*) DNA content and genomic copy number per symbiont cell analysed using VIMPCS. Red arrowhead indicates the means of DNA content and genomic copy number per cell in 46 symbiont cells. (*h*) A high magnification view of the symbiont cell stained with DAPI for VIMPCS analysis, revealing that a signal spot representing a single symbiont cell consists of several small units (white arrowheads). Scale bars in (*d*) 10 µm; (*h*) 2 µm. Data are available in the electronic supplementary material, table S1.

To know the population size of the *C. okutanii* symbiont transmitted via eggs, we first quantified the genomic DNA of the symbiont per individual egg by qPCR. The result indicates that an individual egg of *C. okutanii* carries on average 5220 ± s.d. 1979 molecules of the symbiont genomic DNA (figure 3*a*). Next, we manually counted the cell number of the symbiont per egg stained with DAPI. As is the case in the ISH result, the DAPI signals were detected at the surface of the egg in an approximately oval-shaped cluster (figure 3*b–d*). The data revealed that on average 408 ± s.d. 95 symbiont cells are located on the surface of an individual host egg (figure 3*e*).

These results suggested that the symbiont was polyploid. To confirm this, we directly quantified the DNA content of individual symbiont cells by fluorimetry using VIMPCS [17,18] using eggs from host individual No.5 (figure 3*a*,*e*,*f*) stained with DAPI. The mean DNA content per cell was 8.61 ± s.d. 3.19 Mbp, corresponding to 8.43 ± s.d. 3.12 copies of the *C. okutanii* symbiont genome [3] (figure 3*g*). This was consistent with the calculation from the result of qPCR and cell number counting to be 10.4 ± s.d. 2.58 copies (figure 3*f*) for the symbionts from host individual No.5, and supported the polyploidy of the symbiont. For all five hosts, the calculation from the results of qPCR and cell number counting yielded an average of 12.8 ± s.d. 5.70 genomic copies per cell (figure 3*f*). In observation of the DAPI-stained symbionts at high magnification, we found that a signal spot representing a single symbiont cell consisted of several small units (figure 3*h*). In the VIMPCS analysis, the mean DNA content per this small unit was 1.09 ± s.d. 0.26 Mbp, suggesting that this unit represented a single copy of the symbiont genome (1.02 Mbp [3]). Thus, we conclude that an individual egg carries about 400 polyploid symbiont cells containing approximately 10 genomic copies.

## 4. Discussion

### 4.1. The symbiont transmission mode of vesicomyid clams and its correlation with host development

Although it has been long believed that 'intracellular' symbionts of vesicomyid clams are vertically transmitted between the generations, no conclusive evidence has shown an association of the symbiont with spawned eggs. Here, we have finally revealed that the symbiotic bacteria of *C. okutanii* are associated with the host's egg and are extracellularly attached on the egg plasma membrane. The egg surface is one of the most commonly described routes of symbiont transmission in various insects and some gutless oligochaetes [6,24]. However, this route has been reported only in transmission of extracellular symbionts with very few exceptions [25,26]. Extracellular localization of the symbiont on the surface of the egg in *C. okutanii* may represent a new and unique mechanism of intracellular obligate symbiont transmission.

Previous studies of vesicomyid clams have shown largely congruent branching patterns between the host and symbiont phylogenies, supporting vertical transmission in this taxon [27]. However, Stewart and colleagues [10] have suggested additional horizontal acquisition of symbionts might be involved and proposed some hypothetical mechanisms to explain it. These mechanisms include egg-to-egg transmission, in which symbionts are transmitted between contemporary species with direct contact between symbiont-associated eggs, or between eggs and host tissue [10]. The extracellular localization of the vesicomyid clam symbiont on the egg surface demonstrated in this study may provide a concrete route for this hypothesis, when eggs from distinct species come into physical contact in their natural habitat. In most cases, the vitelline membrane enclosing the eggs with the symbionts attached may block direct contact with other eggs or host tissue, reducing the frequency of the host switching and making such horizontal transmission an occasional and historical event.

In our ISH analysis and TEM observation, symbionts were also detected in the acinal wall cells of the ovary. The acinal wall cells have been proposed in some molluscs to be a site for synthesizing and providing yolk protein. In these cells, vitellogenin, a precursor of yolk protein, is produced and probably transported to the maturing oocytes through the extracellular space [28]. The *C. okutanii* symbiont may be transported from the acinal wall cells to the oocyte through the yolk transport route during oocyte maturation, even though the final localization of the yolk protein (in the oocyte) and the symbiont (on the outer surface of the oocyte) are different. Abundant rough endoplasmic reticulum observed in the cytoplasm of the acinal wall cells in our TEM observation suggests secretory ability of these cells (figure 2*e*). It is also possible that the oocyte as well as the acinal wall cells harbour the symbiont in the cytoplasm from their developmental origin, if they are derived from the same cell lineage. In the oyster *Crassostrea gigas*, it has been suggested that follicle cells and germ cells could have the same origin, arising from the differentiation of primordium gonocytes [29]. Observations of symbionts within the gonadal oocyte in the other vesicomyid species [7,9] suggest that the symbiont may come out of the oocyte and attach to its outer surface at some stage during oocyte maturation. More extensive analysis on the localization of the symbiont during gonadal development and oogenesis will improve our understanding of these possibilities.

In adult vesicomyid clams, symbionts have been observed exclusively in the gill and the ovary. How is this related to the asymmetric localization of the symbiont at the egg vegetal pole as we found in *C. okutanii*? In insects, the asymmetric localization of their endosymbionts in the oocytes has been widely observed. In many cases, the symbionts aggregate at the posterior pole of the egg, guaranteeing symbiont integration into the future germline [1,30,31]. In the oyster *C. gigas*, germplasm contributing to the germline is likely to be localized at the vegetal pole of the eggs [32]. Furthermore, in *Dentalium* scaphopods, a number of bacteria-like bodies were reportedly attached to the outer surface of the egg's vegetal pole, and in later development were visible only on a blastomere, a part of whose descendants contribute endomesoderm, including the gonadal cell line [33–35]. Similarly, *C. okutanii* symbionts at the egg vegetal pole may integrate into the gonadal cell line via this route, either remaining extracellularly throughout the host development, or intracellularly, entering the host cells at some point during development. If this is the case, the symbiont population should be divided into a gonadal cell line and a population that enters the gill during development. It is not clear how the symbionts reach the developing gills and enter the bacteriocytes, or how they exist exclusively in the female gonad. Further embryological studies will help answer these questions.

### 4.2. Population size of the symbiont transmitted via eggs and the evolution of its genome

The *C. okutanii* bacterial symbiont has a smaller genome (1.02 Mbp) than closely related free-living bacteria from the same environment [3]. In general, obligate mutualistic endosymbionts usually possess drastically reduced genomes. This is presumed to be driven by gene loss resulting from a combination of strong genetic drift in small populations undergoing severe bottlenecks during transmission, and less selection pressure to maintain genes necessary for an extracellular lifestyle [6,36].

Here we have shown that an individual egg of *C. okutanii* carries on average 400 symbiont cells and 5200 molecules of symbiont genomic DNA and that each bacterial cell contains on average approximately 10 genomic copies. In the aphid *Acyrthosiphon pisum*, harbouring a vertically transmitted endosymbiont *Buchnera* with a genome size of 0.64 Mbp, the total number of *Buchnera* cells transmitted to each egg has been estimated to be about 1800 [30], although its genomic copy number in each egg has not been reported. Thus, the population size of the *C. okutanii* symbiont transmitted to each egg is relatively very small, which might severely affect the rate of genome reduction in this bacterium. Actually, the genome of the *C. okutanii* symbiont is the smallest reported genome among autotrophs [3,37].

On the other hand, given that the *C. okutanii* symbiont exists extracellularly for part of its life, it probably retain genes necessary to survive in less stable environmental conditions and to re-enter host cells at some point during development. The genes identified in the genome of the *C. okutanii* symbiont for synthesis of lipopolysaccharides (LPS) suggest that it can assemble the LPS structures, which may be related to its ability to live outside of the host cell [38]. Furthermore, it has been shown previously that the *C. okutanii* symbiont in the gill bacteriocyte contains one genomic copy per cell [3]. Multiple genomic copies of the symbiont associated with eggs might be stock for forthcoming cell divisions of the symbionts during embryonic development, when metabolic resources are likely to be limited relative to those in the gill. Alternatively, the polyploidy might slow Muller's ratchet, which otherwise would be serious to small and asexual populations [39,40], provided that multiple genomic copies are retained in the germline over the host's generations, and recombination between these genomic copies takes place as noted previously [40].

The stage of reductive genome evolution in *Calyptogena* symbionts was proposed to be earlier than that in *Buchnera*, since *Calyptogena* symbionts have a lower synonymous mutation value and many large-sized deletions compared with other insect symbiont pairs, such as *Buchnera* strains [37]. Genomic features among *Calyptogena* symbionts may be consistent with the results presented here indicating small population size in transmission characterized by polyploidy and a transient extracellular lifestyle. Our results, taken together with previous genomic analyses, suggest that the *Calyptogena* symbiont is in an intermediate phase of reductive genome evolution.

In this study, we have shown that the symbiotic bacteria of *C. okutanii* are located at the egg's vegetal pole, attached extracellularly on the outer surface of the egg plasma membrane, and that an individual egg carries on average about 400 symbiont cells containing around 10 genomic copies. Here we suggest a possibility that these factors could have opposing impacts on evolution of the symbiont genome. The very small population size of the symbiont during transmission might increase genetic drift, while polyploidy and its transient extracellular lifestyle might slow the rate of genome reduction, although further studies should be performed to evaluate this possibility.

## Supplementary Material

Supplemental_Figures.pdf: Supplemental Figures S1 and S2

## Supplementary Material

Table_S1.xlsx: Supplemental Data table S1

## Data Accessibility

The datasets supporting this article have been uploaded as part of the electronic supplementary material, table S1.
