# Supplementary material for: Surfing the vegetal pole in a small population: extracellular vertical transmission of an 'intracellular' deep-sea clam symbiont
Source: R Soc Open Sci. 2016 May 18;3(5):160130. doi: 10.1098/rsos.160130 (PMC4892456; doi:10.1098/rsos.160130)
Supplement: Supplemental_Figures.pdf: Supplemental Figures S1 and S2 [file rsos160130supp1.pdf]

Supplementary Figures

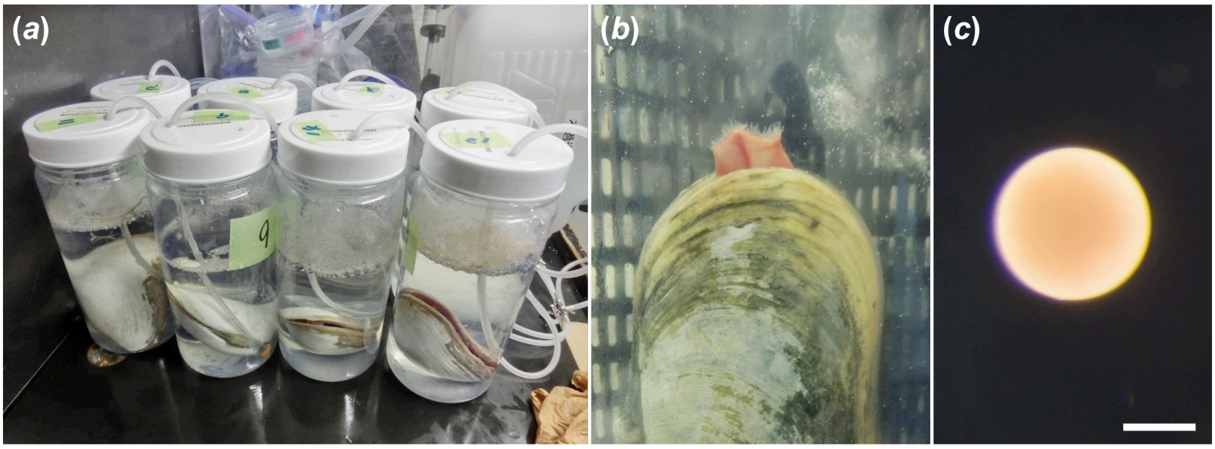

Figure S1. On-board induction of spawning in *C. okutanii*.

(***a***) A snapshot of on-board spawning induction. Immediately after injection of 5-HT, each clam was placed individually in plastic containers holding about 2 L of seawater at 4°C with aeration. (***b***) A clam releasing eggs in a container. (***c***) A spawned egg. Bar, 100 µm.

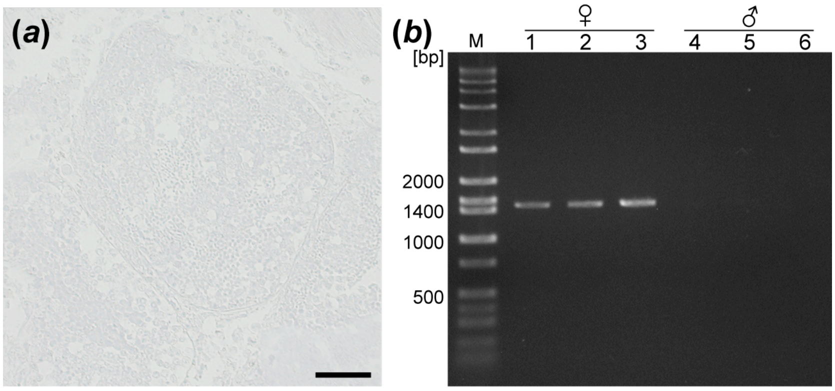

Figure S2. Additional experiments for localization of the symbiont in the gonad.

(***a***) ISH with Cok 16S_1 probe on the testis. Bar, 50 µm. (***b***) PCR for detecting the symbiont in the gonads using primer sets for *16S rRNA* gene of the symbiont. Lanes 1-3: ovaries from three host females. Lanes 4-6: testes from three host males.
